# Recurrence in ileosigmoid knotting

**DOI:** 10.12669/pjms.42.5.15007

**Published:** 2026-05

**Authors:** Mesud Fakirullahoglu, Esra Disci, Rifat Peksoz, Enes Agirman, Sabri Selcuk Atamanalp

**Affiliations:** 1 Mesud Fakirullahoglu, MD Assistant Professor, Department of General Surgery, Erzurum City Hospital, Erzurum, Turkiye; 2 Esra Disci, MD Associate Professor, Department of General Surgery, Faculty of Medicine, Ataturk University, Erzurum, Turkiye; 3 Rifat Peksoz, MD Associate Professor, Department of General Surgery, Faculty of Medicine, Ataturk University, Erzurum, Turkiye; 4 Enes Agirman, MD Assistant Professor, Department of General Surgery, Erzurum City Hospital, Erzurum, Turkiye; 5 Sabri Selcuk Atamanalp, MD Professor, Department of General Surgery, Faculty of Medicine, Ataturk University, Erzurum, Turkiye

**Keywords:** Emergency surgery, Ileosigmoid knotting, Recurrence, Sigmoid volvulus

## Abstract

**Objectives:**

Ileosigmoid knotting (ISK) is a rare but complicated form of ileum volvulus (IV) and sigmoid volvulus (SV). Our aim was to explore the recurrence of ISK, IV, and SV in cases with ISK.

**Methodology:**

The clinical data of Ataturk University, Faculty of Medicine, Department of General Surgery, were evaluated in a partial retrospective (from June 1966 to July 1986) and prospective (from July 1986 to January 2026) consideration. On the other hand, worldwide data were obtained from the last 81 years’ literature (from 1945 to 2026) by electronic search of Web of Science and PubMed databases.

**Results:**

In 59.5-year period, we surgically treated 81 patients with ISK. Although we did not determine any ISK or IV recurrence, early and late SV recurrences were demonstrated in one for each (1.3%, one of living 79 patients and 4.5%, one of followed-up 22 patients, respectively). As worldwide data, early ISK, IV, and SV recurrences were determined one for each except for our one case with early SV recurrence, while no late recurrence was found among limited data. Primary surgery was detorsion alone in all above-mentioned recurrent cases.

**Conclusion:**

Early ISK, IV, and SV recurrences are uncommon outcomes of ISK. However, long-term outcomes are controversial due to the insufficient data. The most important cause of recurrence was conservative surgery including detorsion alone. Emergency or elective definitive surgery including ileum resection or cecopexy (in cases elongated ileum or mobile cecum) and sigmoidopexy or sigmoid colectomy (in cases with sigmoid dolichocolon) may reduce ISK, IV, and SV recurrences in selected patients with ISK.

## INTRODUCTION

Ileosigmoid knotting (ISK) is a complex form of ileum volvulus (IV) and sigmoid volvulus (SV).^1^ Recurrence is a well-defined outcome of SV in patients treated with conservative procedures.^1^,^2^ However, recurrence of ISK, IV, or SV in cases with ISK is a controversial subject.^3^,^4^ Although ISK is a rare disease worldwide, it is relatively common in Eastern Turkey with an incidence of 0.3 patients per 100,000 persons per year.^5^,^6^ According to a data search of the last 81 years of literature (between 1945 and 2026) in Web of Science^7^ and PubMed^8^ databases, among little more than one thousand cases, our series of 81 patients contains the third largest single-center ISK data over the world. In this article, we aimed to investigate the recurrence of ISK, IV, and SV in patients with ISK in both our series and worldwide data.

## METHODOLOGY

In the evaluation of our ISK series treated in Ataturk University, Faculty of Medicine, Department of General Surgery, we utilized combined retrospective (56 patients, 69.1%, between June 1966 and July 1986) and prospective (25 cases, 30.9%, between July 1986 and January 2026) search. Following diagnosis and resuscitation, we applied emergency surgery in all patients. In some patients with viable bowel, we generally preferred detorsion alone or mezosigmoidopexy, while some others treated with bowel resection. On the other hand, patients with bowel gangrene required resection of the gangrenous segment followed by primary anastomosis or stoma. To obtain the worldwide data of the last 81 years’ literature (from 1945 to 2026), we searched Web of Science^7^ and PubMed^8^ databases under the heading of ‘ileosigmoid knotting’.

In the consideration of ISK, IV, and SV recurrence, were used the same diagnostic criteria for primary diseases. Recurrence during the first admission was accepted as ‘early recurrence’, while it was accepted as ‘late recurrence’ when diagnosed at any one time following the discharge. In numerical analysis, we used numbers and percentages for evaluation and comparison.

### Ethical Approval:

We obtained institutional review board from Ethical Committee of Ataturk University Faculty of Medicine (2024/69; dated February 21, 2024). We provided written informed consent from all patients or from their relatives.

## RESULTS

**Fig.1:**
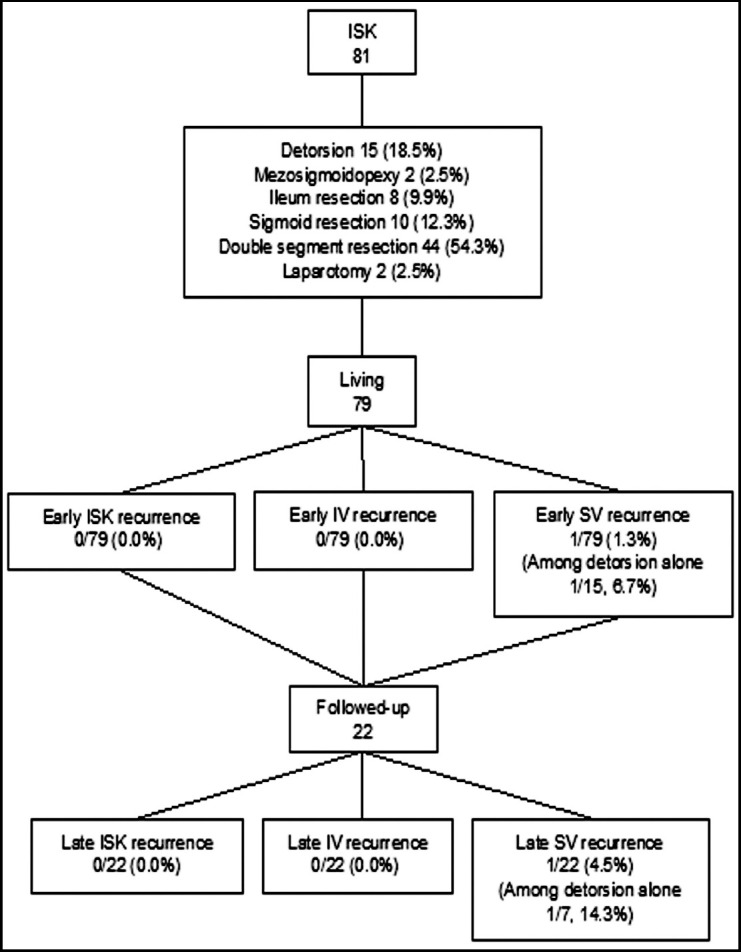
Treatment options and recurrence in our 81 patients with ileosigmoid knotting (ISK: ileosigmoid knotting, IV: ileum volvulus, SV: sigmoid volvulus).

Apart from two patients (2.5%) died during laparotomy, we treated 15 of 81 ISK patients (18.5%) with detorsion alone and two cases (2.5%) with mezosigmoidopexy, while ileum resection was required in eight cases (9.9%), sigmoid resection in 10 (12.3%), and double-segment resection in 44 (54.3%). Fig.1, among 79 living patients (97.5%) with ISK, we could not determine any early ISK or IV recurrence (0.0%), while early SV recurrence was determined in one case (1.3%), whose primary treatment option was surgical detorsion alone (1 of 15 patients with detorsion, 6.7%). On the other hand, among 22 patients (27.8%), in whom follow-up was possible (mean follow-up period: 30.6-year, range: 2-42 years), we had no late ISK or IV recurrence (0.0%), while one patient (4.5%) presented SV recurrence, whose primary treatment option was also surgical detorsion alone (1 of 7 patients with detorsion, 14.3%). In our series, all recurrent SV cases were treated with endoscopic detorsion.

Regarding the worldwide data on ISK, among the last 81 years’ literature (from 1945 to 2026), from the first report by Shepherd et al.^9^ in 1967 to 2026, we found 123 and 101 publications, in Web of Science^7^ and PubMed^8^ databases, respectively. When duplicated, unfindable, or inadequate publications were excluded, we evaluated total 99 papers. As demonstrated in Table-I, according to obtained data, early ISK, IV, and SV recurrences were determined one for each except for our one case with early SV recurrence. Unfortunately, almost all of the publications were about early outcomes of ISK. For this reason, limited information was available regarding the late recurrence. Among them, there was no late ISK, IV, and SV recurrence.^5^,^9^-^18^

**Table-I:** Recurrence in patients with ileosigmoid knotting in different series including 20 or more patients in worldwide literature.

Author	Year	Country	Patient	ISK		IV		SV	

				recurrence		recurrence		recurrence	

				Early	Late	Early	Late	Early	Late
Shepherd9	1967	Uganda	67	-	-	-	-	-	-
Alver et al.10	1993	Turkey	68	-	-	1	-	1	-
Kotisso and Bekele11	2006	Ethiopia	22	-	-	-	-	-	-
Machado et al.12	2009	Worldwide	280	-	-	-	-	-	-
Chalya et al.13	2015	Tanzania	24	-	-	-	-	-	-
Cakir et al.14	2015	Turkey	36	-	-	-	-	-	-
Ooko et al.15	2016	Kenya	61	-	-	-	-	-	-
Mbanje et al.16	2020	Zimbabwe	21	-	-	-	-	-	-
Abebe et al.17	2020	Ethiopia	34	-	-	-	-	-	-
Atamanalp et al.18	2022	Worldwide	923	1	-	-	-	1*	-
Bayleyegn et al.5	2024	Ethiopia	40	-	-	-	-	-	-
Bitewa et al.3	2025	Ethiopia	42	-	-	-	-	-	-
Present series	2026	Turkey	81	-	-	-	-	1*	1

Total				1	-	1	-	2	1

ISK: ileosigmoid knotting, IV: ileum volvulus, SV: sigmoid volvulus,

* One case is dublicated.

## DISCUSSION

When we add the largest worldwide series including 923 cases published in 2022,^18^ the number of reported ISK cases rises up to little more than one thousand in the literature.^7^,^8^ Although recurrence is an ineluctable result in SV patients treated with detorsion alone, recurrence of ISK, IV, or SV is uncommon in patients with ISK.^3^,^5^,^6^,^10^,^18^,^19^

In our 81 case series, there was no early ISK or IV recurrence, while early SV recurrence was seen in one case. Although a relatively limited number of follow up was an important limitation of the present series, we demonstrated that ISK and IV recurrences are rare outcomes of ISK, while SV recurrence is relatively more frequent. However, when compared with that of SV, ISK or IV are more dangerous clinical entities with more difficult diagnosis and heavier prognosis. Although rare, to prevent or reduce all kinds of recurrence must be one of the main targets in the treatment of ISK.

Among worldwide data on patients with ISK, only one early ISK recurrence was reported in the largest multicenter series over the world.^18^ The first procedure was surgical detorsion alone of this patient, who was treated with surgical detorsion again. Similarly, only one early IV recurrence was presented by Alver et al,^10^ in which patient, the primary treatment option was also surgical detorsion alone. In this case, the causative factor was an adhesive band, which problem was evaluated by emergency surgery including detorsion and removal of the adhesion. Regarding SV recurrence, we meet with two patients with early and late recurrences one for each in our series. Additionally, Alver et al,^10^ presented a case with early SV recurrence. Interestingly, in all of above-mentioned SV recurrences, the primary surgical procedure was detorsion alone and they were treated with endoscopic detorsion.

Because of the rarity of ISK, IV, and SV recurrences following ISK, management strategies become crucial, in which the main rules of the primary disease are generally valid.^10^,^18^ In recurrent ISK and IV, surgery is the unique treatment way. In cases with gangrenous bowel, emergency surgery including resection of the gangrenous segments is essential. In such patients, intestinal continuity may be obtained by primary anastomosis in well-conditioned and non-elderly people, while stoma may be lifesaving in bad-conditioned and elderly cases. Regarding recurrent SV, gangrenous cases requires emergency surgery including above-mentioned basic rules.^3^,^5^,^20^,^21^ However, the treatment of cases with viable bowel is controversial in recurrent ISK and IV, in whom alternative recurrence-reducing procedures mentioned below may be applied during surgical treatment. Additionally, endoscopic detorsion may be a good bet in SV.^3^,^5^,^19^,^22^,^23^

The prognosis is relatively poor (up to 60% mortality and 80% morbidity rates) in primary ISK.^24^,^25^ Although there is not enough data to evaluate the prognosis of recurrence in patients with ISK, recurrence may aggravate the prognosis particularly in cases with advanced age, comorbid disease, and late admission.^7^,^8^ For this reason, to prevent recurrence of such entities are as important as their treatment. In our opinion, in selected non-gangrenous cases with ISK, particularly in children and young ones, whose life expectancy are long, one of recurrence-reducing procedures may be performed in the first operation. In cases with mobile cecum or elongated ileum, which are the main pathophysiological predisposing factors in the development of ISK and IV, cecopexy or ileum resection may reduce the recurrence. Similarly, in cases with sigmoid dolichosigmoid, which is the main anatomical prerequisite in the development of SV, sigmoidopexy or sigmoid colectomy may reduce the recurrence.^21^,^23^,^25^ As an alternative, recurrence-reducing procedures may be used in elective circumstances, particularly in index admission or two-three weeks later.^21^,^22^,^24^

### Limitations:

Although we evaluated a relatively large ISK series including both our series and worldwide data, our findings on early ISK recurrence largely depended on retrospective evaluation. Additionally, despite a comprehensive search, we could not get enough data on late ISK recurrence due to the insufficient data. However, large-series prospective evaluation on early and late ISK recurrences require hundreds of ISK cases, which may be possible if and only in several decades.

## CONCLUSIONS

Early recurrences of ISK, IV, and SV are rare outcomes of surgical treatment of ISK, while the incidence of late recurrences are not well known. However, it is clear that, conservative surgery is the unique cause of recurrence in ISK. Elective ileum resection or cecopexy (in cases elongated ileum or mobile cecum) and sigmoidopexy or sigmoid colectomy (in cases with sigmoid dolichocolon) performed during the first operation may reduce ISK, IV, and SV recurrences in ISK.

### Author’s Contribution:

**MF:** Data collection, manuscript writing.

**ED RP EA :** Data collection, revision of the final draft.

**SSA:** Data collection, manuscript writing and is responsible and accountable for the accuracy or integrity of the work.

All authors have read and approved the final version.

**Retraction Announcement**

The following manuscript has been retracted today May 4th, 2026 from one of our past issue May–June 2002 on the request of the authors due to some problemes in data integrity and statistical analysis that undermine the reliability of the study results. - ***Editor***

***Retraction in:*** Li L, Chen Q. Application and accuracy analysis of three-dimensional transvaginal ultrasound in the diagnosis of endometrial lesions in postmenopausal women. Pak J Med Sci. 2022;38(5):1205-1209. doi: 10.12669/pjms.38.5.5331
